# Role of systemic immune-inflammation index in patients treated with salvage radical prostatectomy

**DOI:** 10.1007/s00345-021-03715-4

**Published:** 2021-05-17

**Authors:** Pawel Rajwa, Victor M. Schuettfort, Fahad Quhal, Keiichiro Mori, Satoshi Katayama, Ekaterina Laukhtina, Benjamin Pradere, Reza Sari Motlagh, Hadi Mostafaei, Nico C. Grossmann, Andreas Aulitzky, Andrzej Paradysz, Pierre I. Karakiewicz, Harun Fajkovic, Kristin Zimmermann, Axel Heidenreich, Paolo Gontero, Shahrokh F. Shariat

**Affiliations:** 1 Department of Urology, Comprehensive Cancer Center, Medical University Vienna, Vienna General Hospital, Währinger Gürtel 18-20, 1090 Vienna, Austria; 2 Department of Urology, Medical University of Silesia, Zabrze, Poland; 3 Department of Urology, University Medical Center Hamburg-Eppendorf, Hamburg, Germany; 4 Department of Urology, King Fahad Specialist Hospital, Dammam, Saudi Arabia; 5 Department of Urology, The Jikei University School of Medicine, Tokyo, Japan; 6 Department of Urology, Okayama University Graduate School of Medicine, Dentistry and Pharmaceutical Sciences, Okayama, Japan; 7 Institute for Urology and Reproductive Health, Sechenov University, Moscow, Russia; 8 Men’s Health and Reproductive Health Research Center, Shahid Beheshti University of Medical Sciences, Tehran, Iran; 9 Research Center for Evidence Based Medicine, Tabriz University of Medical Sciences, Tabriz, Iran; 10 Department of Urology, University Hospital Zurich, Zurich, Switzerland; 11 Cancer Prognostics and Health Outcomes Unit, University of Montreal Health Centre, Montreal, Canada; 12 Karl Landsteiner Institute of Urology and Andrology, Vienna, Austria; 13 Department of Urology, Federal Armed Services Hospital Koblenz, Koblenz, Germany; 14 Department of Urology, University Hospital Cologne, Cologne, Germany; 15 Division of Urology, Department of Surgical Sciences, San Giovanni Battista Hospital, University of Studies of Torino, Turin, Italy; 16 Department of Urology, Weill Cornell Medical College, New York, NY USA; 17 Department of Urology, University of Texas Southwestern, Dallas, TX USA; 18 Department of Urology, Second Faculty of Medicine, Charles University, Prague, Czech Republic

**Keywords:** SII, Biomarkers, Salvage radical prostatectomy, Prostate cancer, Survival

## Abstract

**Purpose:**

To examine the predictive and prognostic value of preoperative Systemic Immune-inflammation Index (SII) in patients with radio-recurrent prostate cancer (PCa) treated with salvage radical prostatectomy (SRP).

**Materials and methods:**

This multicenter retrospective study included 214 patients with radio-recurrent PCa, treated with SRP between 2007 and 2015. SII was measured preoperatively (neutrophils × platelets/lymphocytes) and the cohort was stratified using optimal cut-off. Uni- and multivariable logistic and Cox regression analyses were performed to evaluate the predictive and prognostic value of SII as a preoperative biomarker.

**Results:**

A total of 81 patients had high preoperative SII (≥ 730). On multivariable logistic regression modeling, high SII was predictive for lymph node metastases (OR 3.32, 95% CI 1.45–7.90, *p* = 0.005), and non-organ confined disease (OR 2.55, 95% CI 1.33–4.97, *p* = 0.005). In preoperative regression analysis, high preoperative SII was an independent prognostic factor for cancer-specific survival (CSS; HR 10.7, 95% CI 1.12–103, *p* = 0.039) and overall survival (OS; HR 8.57, 95% CI 2.70–27.2, *p* < 0.001). Similarly, in postoperative multivariable models, SII was associated with worse CSS (HR 22.11, 95% CI 1.23–398.12, *p* = 0.036) and OS (HR 5.98, 95% CI 1.67–21.44, *p* = 0.006). Notably, the addition of SII to preoperative reference models improved the C-index for the prognosis of CSS (89.5 vs. 80.5) and OS (85.1 vs 77.1).

**Conclusions:**

In radio-recurrent PCa patients, high SII was associated with adverse pathological features at SRP and survival after SRP. Preoperative SII could help identify patients who might benefit from novel imaging modalities, multimodal therapy or a closer posttreatment surveillance.

**Supplementary Information:**

The online version contains supplementary material available at 10.1007/s00345-021-03715-4.

## Introduction

Radiation therapy is an effective therapy for localized prostate cancer (PCa) with durable local control [1, 2]. After primary radiation, however, up to 50% of patients experience biochemical recurrence (BCR), which is associated with subsequent risk of metastasis and PCa-specific death [3–5]. While some of these patients develop distant recurrence, a large proportion would benefit from effective local salvage therapy [2]. One of them is salvage radical prostatectomy (SRP), which offers a possibility of cure, and is associated with 53% 5-year recurrence-free survival (RFS) and over 70% 10-year cancer-specific survival (CSS) [2, 6]. These favorable long-term outcomes after SRP comes at the cost of potentially significant adverse events, including incontinence, although improvement has been reported in recent years [2, 6–8]. This risk for adverse events could be acceptable if a cure or long-term remission can be achieved. The current prediction of outcomes based on clinicopathologic is, however, suboptimal. Considering the growing interest of salvage modalities in radio-recurrent PCa, there is an urgent need to improve risk stratification to guide treatment decision making with respect to radical, focal or systemic therapy [2, 5, 6]. The Systemic Immune-inflammation Index (SII) is a novel biomarker, which combines three immune cell counts into a simple formula: neutrophils × platelets/lymphocytes [9]. Through the incorporation of single components of well-known prognostic biomarkers in urologic oncology, neutrophil-to-lymphocyte ratio (NLR) and platelet-to-lymphocyte ratio (PLR), the SII comprehensively depicts the cancer-related inflammatory burden [9, 10].

So far, the prognostic ability of the SII prognostic has been confirmed in the context of castration-resistant prostate cancer (CRPC) treated with systemic therapy but no data exist on SII in radio-recurrent PCa [9]. Therefore, we aimed to analyze the predictive and prognostic value of SII in a large cohort of radio-recurrent PCa patients who underwent SRP.

## Materials and methods

We retrospectively reviewed data from five academic centers including patients with clinical non-metastatic radiation-recurrent PCa treated with SRP between 2007 and 2015. Local institutional review board approved this study (No. 1104011637). The database and follow-up have previously been described in detail [11, 12]. In general, patients were treated with primary radiation therapy, which included either brachytherapy or external beam radiation therapy (EBRT) or combination techniques (EBRT and brachytherapy, EBRT and intensity-modulated radiation therapy, or EBRT and three-dimensional conformal radiation therapy). BCR after RT was defined as PSA ≥ 2 ng/ml greater than the nadir (Phoenix criteria) [13]. Before SRP, all patients underwent confirmatory biopsy. No patient was diagnosed with imaging-detected metastases before SRP. All patients underwent SRP with pelvic lymph node dissection. PCa staging and grading were performed according to the 2007 American Joint Committee on Cancer Tumor Nodes Metastasis (TNM) staging system and 2006 Gleason grading consensus, respectively [14]. All prostate specimens were examined by dedicated genitourinary pathologists at all centers. Non-organ confined disease was defined as pT ≥ 3 and/or pN ≥ 1; adverse pathology was defined as pT ≥ 3 and/or pN ≥ 1 and/or GS ≥ 8 and/or positive surgical margins.

### Follow-up

Patients generally underwent PSA testing and physical examination every 3 months within the first 2 years and every 6 months thereafter. We defined post-SRP BCR as PSA ≥ 0.2 ng/mL. No patients received adjuvant androgen deprivation therapy (ADT) before the diagnosis of BCR. Distant metastases were identified using radiologic imaging. The cause of death was retrieved from medical records and/or death certificates. For PCa-specific death, only men with known recurrence after SRP, who had documented metastatic PCa, and who had PCa listed in the death certificate were considered to have died of PCa. We calculated follow-up from the date of RP to the date of death or last follow-up visit.

### Systemic immune-inflammation index (SII)

SII data were retrieved from pre-SRP complete blood count and calculated as follows: neutrophils absolute count x platelets absolute count divided by lymphocytes absolute count. Preoperative SII cut-off point was determined by Receiver Operating Characteristics (ROC) curve analysis using the Youden index for cancer-specific survival (CSS). In summary, the Youden index provides the optimal cut-off from a continuous variable by showing the score that offers the best tradeoff between sensitivity and specificity. Using this score, the overall population was divided into two separate SII groups (low vs. high).

### Statistical analyses

Associations between SII values and patients' clinicopathologic features were evaluated using the Wilcoxon rank-sum test for continuous variables and chi-square test of independence or Fisher's exact test for categorical variables, as appropriate. Univariable and multivariable logistic regression analyses tested the association of SII with adverse pathologic findings. The models’ predictive accuracy was analyzed using receiver operating characteristics (ROC) curves and calculating the derived area under the curve (AUC). AUCs were statistically compared using DeLong’s test. Kaplan–Meier estimates with log-rank testing were used to depict the association between preoperative SII and survival outcomes. Pre- and posttreatment univariable and multivariable Cox regression analyses analyzed the association of SII with BCR-free survival (BFS), metastasis-free survival (MFS), CSS, and overall survival (OS). *p* value of < 0.05 was considered as the threshold of statistical significance. All tests were two sided. Analyses were performed using R Version 4.0 (R Foundation for Statistical Computing, Vienna, Austria, 2020).

## Results

Overall, 214 patients with radio-recurrent PCa, who underwent SRP were included in our analyses. According to the optimal cut-off of  ≥ 730, 81 patients were categorized to have high preoperative SII. Clinical and pathological features stratified by SII are presented in Table 1. Most of the patients had mild-to-severe concomitant diseases (83% ASA 2–3), however, there were no significant differences between patients with low and high SII. There were significant differences between patients with low and high SII values in SRP GS, positive surgical margins, extracapsular extension, and lymph node metastases at SRP. There were no differences between patients with low and high preoperative SII with regards to radiation therapy type, biopsy (pre-SRP) GS, PSA level, age, and complications as assessed using Clavien–Dindo classification.

**Table 1:** Clinicopathologic features of 214 radio-recurrent patients treated with SRP for radio-recurrent PCa

Characteristic	Overall	Cohort stratified by SII		
	*N* = 214	Low, *N* = 133	High, *N* = 81	*p* value
Age at SRP (IQR)	69 (64, 72)	69 (64–73)	69 (64–72)	> 0.9
ASA status (%)				0.11
1	36 (17)	22 (17)	14 (17)	
2	113 (53)	64 (48)	49 (60)	
3	65 (30)	47 (35)	18 (22)	
BMI (IQR)	24 (24–27)	24 (24–27)	24 (24–27)	0.4
Radiation therapy type				0.3
EBRT	167 (78%)	101 (76%)	66 (81%)	
Brachytherapy	39 (18%)	25 (19%)	14 (17%)	
EBRT + Brachytherapy	8 (3.7%)	7 (5.3%)	1 (1.2%)	
PSA median (IQR)	3.8 (2.1–6.5)	3.9 (2.3–6.4)	3.7 (1.7–6.7)	0.6
Pre-SRP biopsy GS (%)				0.5
GS 6	48 (22)	32 (24)	16 (20)	
GS 7	104 (49)	68 (51)	36 (44)	
GS 8	32 (15)	18 (14)	14 (17)	
GS 9	15 (7.0)	8 (6.0)	7 (8.6)	
GS 10	15 (7.0)	7 (5.3)	8 (9.9)	
Clinical staging (%)				0.2
cT1	99 (46)	64 (48)	35 (43)	
cT2	84 (39)	54 (41)	30 (37)	
cT ≥ 3	30 (14)	14 (11)	16 (20)	
SRP GS (%)				**0.001**
GS 6	14 (6.5)	11 (8.3)	3 (3.7)	
GS 7	114 (53)	83 (62)	31 (38)	
GS 8	43 (20)	20 (15)	23 (28)	
GS 9	30 (14)	14 (11)	16 (20)	
GS 10	13 (6.1)	5 (3.8)	8 (9.9)	
PSM (%)	43 (20)	20 (15)	23 (28)	**0.029**
pT3a (%)	92 (43)	49 (37)	43 (53)	**0.029**
pT3b (%)	67 (31)	36 (27)	31 (38)	0.12
pN ≥ 1 (%)	40 (19)	15 (11)	25 (31)	** < 0.001**
OR (IQR)	198 (150–233)	180 (150–235)	200 (170–220)	0.7
EBL (IQR)	600 (350–900)	650 (400–1000)	600 (350–885)	0.3
Clavien–Dindo complication (%)				0.079
1	21 (9.8)	9 (6.8)	12 (15)	
2	167 (78)	110 (83)	57 (70)	
3	26 (12)	14 (11)	12 (15)	

*ASA* American Society of Anesthesiologists; *BMI* body mass index; *EBL* estimated blood loss; *EBRT* external beam radiation therapy; *GS* Gleason score; *OR* operating time; *PSA* prostate-specific antigen; *PSM* positive surgical margin; *SRP* salvage radical prostatectomy; *SII* Systemic Immune-inflammation Index

Statistics presented: *n* (%); Median (IQR)

Statistical tests performed: Wilcoxon rank-sum test; chi-square test of independence; Fisher's exact test

Significance bold values are *p* < 0.05

In univariable logistic regression analyses, high preoperative SII was associated with higher rates of pT ≥ 3 disease (odds ratio [OR] 1.94, HR 1.10–3.41, *p* = 0.021), lymph node metastasis (OR 3.51, 95% CI 1.72–7.18, *p* = 0.001), non-organ confined disease (OR 2.50, 95% CI 1.40–4.45, *p* = 0.002) and adverse pathology (OR 2.27, 95% CI 1.27–4.07, *p* = 0.006) (Supplementary Table I). In multivariable models that adjusted for the effect of the established clinicopathologic variables (Table 2), SII remained an independent predictive risk factor for lymph node metastasis (OR 3.32, 95% CI 1.45–7.90, *p* = 0.005), non-organ confined disease (OR 2.55, 95% CI 1.33–4.97, *p* = 0.005) and adverse pathology (OR 2.20, 95% CI 1.15–4.33, *p* = 0.019). The incorporation of preoperative SII into predictive reference models, comprising age, biopsy GS, preoperative PSA, and cT stage, did not significantly improve their accuracy with respect to the AUC for adverse pathological findings (Table 2).

**Table 2:** Multivariable logistic regression analyses assessing the association of SII with adverse surgical features in 214 radio-recurrent PCa treated with SRP

Characteristic	*N*	pT ≥ 3			Lymph node metastasis (pN ≥ 1)			Non-organ confined disease			Adverse pathology		
		OR	95% CI	*p* value	OR	95% CI	*p* value	OR	95% CI	*p*-value	OR	95% CI	*p*-value
SII (high vs. low)	214	1.74	0.94–3.22	0.08	3.32	1.45–7.90	**0.005**	2.55	1.33–4.97	**0.005**	2.20	1.15–4.33	**0.019**
Age	214	1.03	0.98–1.08	0.21	0.99	0.92–1.06	0.72	1.04	0.99–1.10	0.09	1.04	0.99–1.09	0.17
Biopsy GS	214	1.26	0.94–1.69	0.12	2.38	1.65–3.54	** < 0.001**	1.53	1.12–2.13	**0.010**	1.66	1.20–2.35	**0.003**
PSA	206	1.10	1.01–1.19	**0.02**	1.11	1.02–1.22	**0.022**	1.18	1.08–1.32	**0.001**	1.17	1.06–1.31	**0.002**
cT													
T1	99	Ref	Ref		Ref	Ref		Ref	Ref		Ref	Ref	
T2	84	1.52	0.82–2.83	0.19	0.57	0.22–1.43	0.24	1.58	0.83–3.04	0.17	1.58	0.83–3.05	0.16
T3	30	2.72	0.99–7.46	0.05	0.75	0.20–2.44	0.64	2.31	0.81–7.31	0.13	3.67	1.18–14.0	**0.035**
		AUC (full model): 0.692 AUC (model without SII): 0.690 *p* = 0.914			AUC (full model): 0.821 AUC (model without SII): 0.767 *p* = 0.055			AUC (full model): 0.748 AUC (model without SII): 0.736 *p* = 0.540			AUC (full model): 0.753 AUC (model without SII): 0.746 *p* = 0.664		

Non-organ confined diseases (pT ≥ 3 and/or pN ≥ 1); Adverse pathology (pT ≥ 3 and/or pN ≥ 1 and/or GS ≥ 8 and/or PSM)

*CI* confidence interval; *DRE* digital rectal examination; *GS* Gleason Score; *OR* odds ratio; *PSA* prostate-specific antigen; *SRP* salvage radical prostatectomy; *SII* Systemic Immune-inflammation Index

**Fig. 1:**
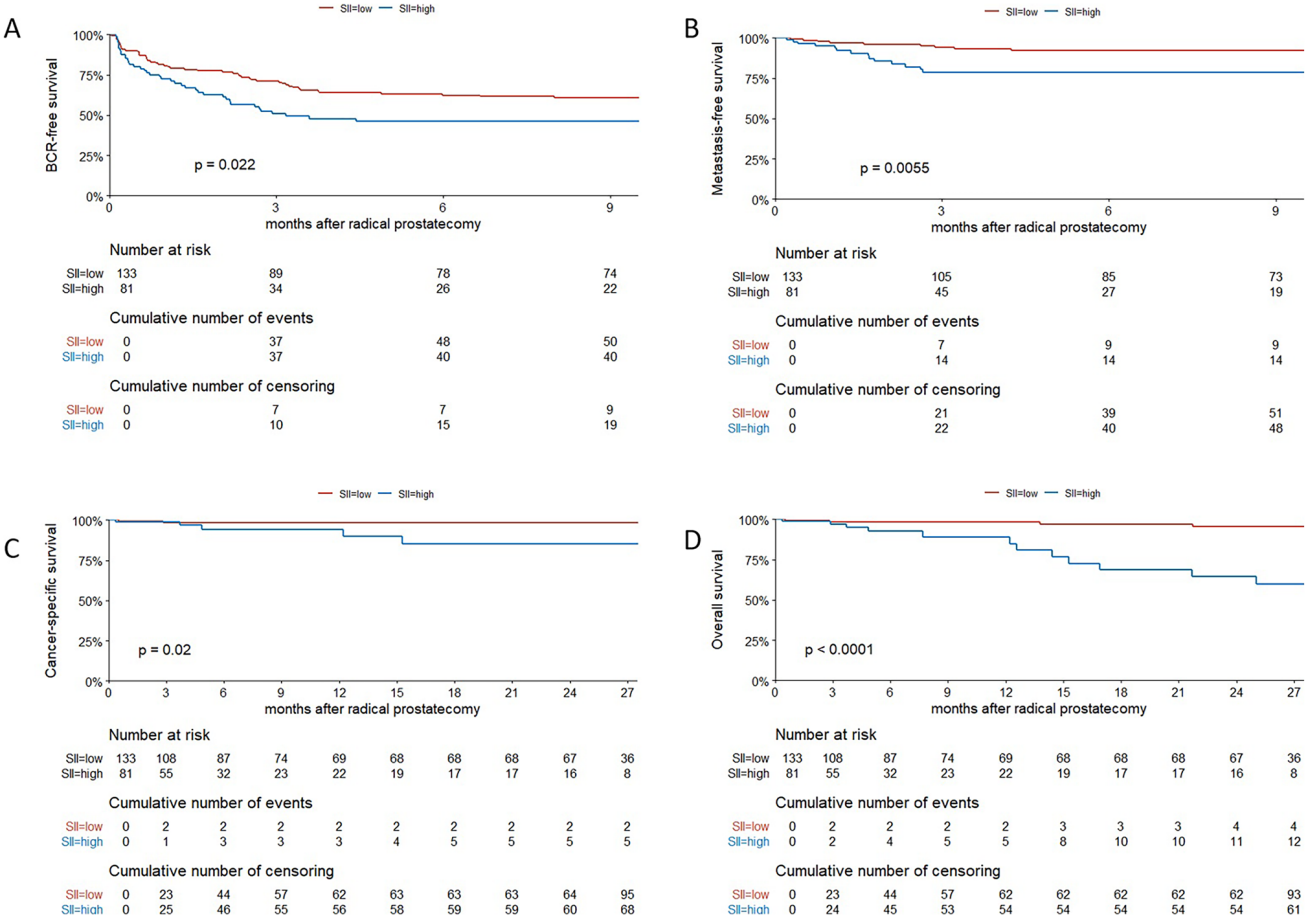
Kaplan–Meier analysis stratified by SII levels for 214 patients treated with SRP for radio-recurrent PCa: **a** for BFS, **b** for MFS, **c** for CSS, **d** for OS

The median follow-up was 25.3 (interquartile range [IQR], 15–28.5) months; 90 (42%) patients experienced BCR, 23 (11%) developed metastases, 7 (3.3%) died from PCa, and 18 (8.4%) died from any cause. On Kaplan–Meier analyses, BFS, MFS, CSS, and OS were worse in patients with high preoperative SII compared to those with low SII (Fig. 1, *p* < 0.05 for all outcomes). On univariable Cox regression analyses, high SII was associated with BCR (HR 1.62, 95% CI 1.07–2.46, *p* = 0.024), MFS (HR 3.09, 95% 1.34–7.17, *p* = 0.008), CSS (HR 5.70, 95% CI 1.09–29.80, *p* = 0.039), and OS (HR 6.21, 95% CI 2.30–16.76, *p* < 0.001). In the preoperative multivariable regression models, high preoperative SII was an independent prognostic factor for CSS (HR 10.7, 95% CI 1.12–103, *p* = 0.039) and OS (HR 8.57 2.7–27.2, *p* < 0.001), but not BCR (HR 1.39, 95% CI 0.89–2.18, *p* = 0.15) or MFS (HR 2.09, 95% CI 0.81–5.40, *p* = 0.129) (Table 3). Similarly, in postoperative multivariable models, high SII was an independent prognostic factor for CSS (HR 22.11, 95% CI 1.23–398.12, *p* = 0.036) and OS (HR 5.98, 95% CI 1.67–21.44, *p* = 0.006) (Supplementary Table II). Incorporation of SII to reference preoperative models, comprising age, biopsy GS, PSA, and cT stage, resulted in the highest improvement of the discrimination ability for the prognosis of MFS (change of C-index of 5%), CSS (change of C-index of 10%), and OS (change of C-index of 9%). In postoperative reference models, the inclusion of SII did not provide meaningful improvement to the C-index for any outcome (change of C-Index ≤ 4.2% for all outcomes).

**Table 3:** Preoperative multivariable Cox regression analyses assessing the association of SII with BFS, MFS, CSS, and OS in 214 radio-recurrent PCa treated with SRP

Characteristic	BFS			MFS			CSS			OS		
	HR	95% CI	*p* value	HR	95% CI	*p* value	HR	95% CI	*p*-value	HR	95% CI	*p*-value
Multivariable preoperative models												
SII (high vs low)	1.39	0.89–2.18	0.147	2.09	0.81–5.40	0.129	10.70	1.12–103	**0.039**	8.57	2.70–27.2	** < 0.001**
Age	1.02	0.99–1.06	0.231	1.02	0.94–1.11	0.642	0.91	0.76–1.08	0.292	1.05	0.95–1.15	0.337
Biopsy GS	1.31	1.07–1.61	**0.008**	2.02	1.38–2.96	** < 0.001**	2.83	1.16–6.88	**0.022**	2.21	1.22–4.01	**0.009**
PSA before SRP	1.02	1.00–1.05	**0.043**	1.05	1.01–1.09	**0.009**	1.05	0.98–1.12	0.222	1.01	0.96–1.06	0.738
cT												
T1	Ref	Ref		Ref	Ref		Ref	Ref		Ref	Ref	
T2	1.41	0.88–2.28	0.157	1.36	0.43–4.28	0.599	1.34	0.19–9.61	0.769	2.14	0.73–6.29	0.169
T3	1.54	0.80–2.95	0.194	2.72	0.79–9.41	0.114	1.09	0.08–14.3	0.947	1.47	0.28–7.57	0.647
	C-index (full model): 67.8 C-index (without SII): 66.2			C-index (full model): 84.1 C-index (without SII): 80.1			C-index (full model): 89.5 C-index (without SII): 80.5			C-index (full model): 85.1 C-index (without SII): 77.1		

*BFS* biochemical recurrence-free survival; *CI* confidence interval; *CSS* cancer-specific survival, CSS; *GS* Gleason score; *HR* hazard ratio; *MFS* metastasis-free survival; *OS* overall survival; *PSA* prostate-specific antigen; *PSM* positive surgical margin; *SRP* salvage radical prostatectomy; *SII* systemic immune-inflammation Index

Significance bold values are *p* < 0.05

## Discussion

Local salvage therapy for radio-recurrent PCa is hampered by the accurate identification of localized versus systemic disease at the time of BCR after primary radiation with curative intent [6, 15]. Current tolls fall short to their predictive accuracy to help guide in clinical decision making towards local salvage versus systemic therapy in this setting. Biomarkers that can capture the inherent biological aggressiveness of the tumor, as well as the host response, may help overcome the current staging and prognosis challenges [4, 16, 17].

In our study, we found that preoperative SII predicts adverse pathologic findings at SRP and prognosticates survival outcomes in a large, multicenter cohort of patients treated with SRP for radio-recurrent PCa. Our results suggest that high SII can be used as a clinical guide to predict the probability of lymph node involvement, non-organ confined disease, and adverse pathology at SRP. In addition, high SII can be considered as a valuable prognostic factor for CSS and OS in radio-recurrent PCa patients treated with SRP. Notably, the incorporation of SII into the preoperative predictive models resulted in a clinically relevant increase of their predictive accuracy, especially with respect to CSS and OS.

No prior studies examined the role of SII in radio-recurrent PCa patients undergoing SRP. Our results indicate that patients with high SII had over three-time higher risk of being diagnosed with pathologically confirmed lymph node metastases and were over twice likely to harbor non-organ confined disease or adverse pathology. Also, the model including SII reached over 80% accuracy for the prediction of lymph node metastasis at SRP. These findings suggest that patients with higher SII levels should undergo more detailed imaging such as prostate-specific membrane antigen (PSMA) positron emission tomography (PET)/X-ray computed tomography (CT). Furthermore, patients with high SII could benefit from a more extensive approach at SRP, which includes extended lymph node dissection or re-irradiation or systemic therapy if surgery might be not technically feasible. Contrary, for patients with lower preoperative SII valuable option, may be focal therapy, which is associated with lower toxicity [2]. In PCa, the inflammatory burden has previously been linked to carcinogenesis and progression [18]. Furthermore, radiation therapy itself is known to trigger inflammatory (immune system) responses [19, 20]. All immune cells, which are components of SII, play a pivotal role in cancer response and cancer-related inflammation [10, 21–24]. Cancer cells facilitate pro-tumorigenic polarization of neutrophils, which modulate the cancer microenvironment and other immune cells to promote tumor development [25]. Platelets have been suggested to contribute to tumor angiogenesis and metastasis [26]. A decreased number of lymphocytes may be a result of their cancer inhibition and is associated with impaired response to carcinogenesis [27, 28]. As a result, SII could serve as comprehensive biomarkers of inflammatory burden in radio-recurrent PCa.

In the case of primary radical prostatectomy, patients with adverse surgical features would undergo adjuvant radiation, but very little is known if (and how) these adverse features at SRP impact distant outcomes, and therefore, how to manage patients with locally advanced disease. Notably, in our cohort, high preoperative SII was a valuable, independent risk stratification tool for the two most important outcomes—CSS and OS. Despite the low number of these events, which are the likely cause for high hazard ratios and broad 95% confidence intervals, the association was robust, and SII enabled a clinically relevant increase of the accuracy of preoperative reference models. In this context, high preoperative SII has two important clinical implications. First, patients with high SII might be considered for other treatment modalities such as systemic therapy, as their clinical benefit of SRP is low. Second, if treated with SRP, these patients should undergo more scrutinous surveillance after SRP. In PCa, SII prognostic value was only evaluated in the context of CRPC patients treated with systemic therapy [9, 21, 29]. In a study of Man et al., high SII (> 535) was associated with OS in the multivariable model (HR 2.133, 95% CI 1.163–3.913, *p* = 0.014) [30]. Lolli et al. found that high SII (≥ 535) was an independent prognostic factor for OS in CRPC patients treated with abiraterone (HR 2.08, 95% CI 1.48–2.92, *p* < 0.01) [29]. Furthermore, in another study, analyzing 104 patients with metastatic CRPC treated with sequential therapy, SII (≥ 200) prognosticated worse OS (HR 9.6, 95% CI 4.7–19.5, *p* < 0.01) and progression-free survival (HR 17.4, 95% CI 9.2–33.0, *p* < 0.01) [31]. This is contradictory to the recent study of Stangl-Kremser et al. who did not find a significant association between SII > 200 and overall survival in the CRPC cohort treated with docetaxel (HR 1.0, 95% CI 0.9–1.0, *p* = 0.06) [21]. The association between and SII was also reported in other solid tumors [23, 32]. For example, Hu et al. analyzed 646 non-metastatic renal cell carcinoma patients treated with nephrectomy and found high SII (> 529) as an independent predictor of CSS (HR = 2.17, 95% CI 1.33–3.55, *p* = 0.002) and OS (HR = 2.26, 95% CI 1.44–3.54, *p* < 0.001) [32]. Also, in a recent meta-analysis of eight studies, Zhang et al. determined high SII as a prognostic factor for worse OS (HR = 1.79, 95% CI 1.33–2.42, *p* < 0.001) in breast cancer patients [23].

Our study has several limitations. This is a retrospective, multicenter study without central pathology examination and modest follow-up. Besides, patients were initially treated with various radiation therapy modalities and operated in multiple centers, and therefore surgical techniques and experiences could differ. Also, centers did not provide details on complications and concomitant diseases, but reported scores based on validated classifications (e.g. Clavien-Dindo and ASA). Furthermore, the SII level might have been biased by the presence of an autoimmune disease or chronic medical condition that can affect SII levels. Also, patients did not undergo PSMA PET-CT imaging for staging, which could result in the inclusion of patients with undetected metastases. Despite these flaws, we presented the first study, which comprehensively analyzed the role of SII in radio-recurrent PCa treated with SRP. Considering the paucity of available biomarkers in radio-recurrent PCa managed surgically, we believe our study provided substantial input to this field. Further studies with a prospective design are needed for validation of these results.

## Conclusions

In radio-recurrent PCa patients, high SII was associated with adverse pathological features at SRP and survival after SRP. Preoperative SII could help identify patients who might benefit from novel imaging modalities, multimodal therapy or a closer posttreatment surveillance. Moreover, SII could improve the accuracy of currently utilized preoperative prognostic factors for CSS and OS. Further studies with a prospective design are needed for validation of these results.

## Supplementary Information

Below is the link to the electronic supplementary material.

Supplementary file1 (DOCX 16 KB)

Supplementary file2 (DOCX 23 KB)
