# Supplementary material for: Role of systemic immune-inflammation index in patients treated with salvage radical prostatectomy
Source: World J Urol. 2021 May 17;39(10):3771–9. doi: 10.1007/s00345-021-03715-4 (PMC8521581; doi:10.1007/s00345-021-03715-4)
Supplement: Supplementary file 1 — Supplementary file1 (DOCX 16 KB) [file 345_2021_3715_MOESM1_ESM.docx]

|  | pT≥3 | | | Lymph node metastasis (pN≥1) | | | Non-organ confined disease | | | Adverse pathology | | |
| --- | --- | --- | --- | --- | --- | --- | --- | --- | --- | --- | --- | --- |
| Characteristic | OR | 95% CI | p-value | OR | 95% CI | p-value | OR | 95% CI | p-value | OR | 95% CI | p-value |
| SII (high vs. Low) | 1.94 | 1.10-3.41 | 0.02 | 3.51 | 1.72-7.18 | <0.01 | 2.50 | 1.40-4.45 | <0.01 | 2.27 | 1.27-4.07 | <0.01 |
| Age | 1.04 | 1.00-1.09 | 0.04 | 1.03 | 0.98-1.09 | 0.28 | 1.06 | 1.01-1.10 | 0.01 | 1.05 | 1.01-1.10 | 0.02 |
| Biopsy GS | 1.48 | 1.14-1.93 | <0.01 | 2.31 | 1.68-3.17 | <0.01 | 1.76 | 1.31-2.35 | <0.01 | 1.90 | 1.39-2.58 | <0.01 |
| PSA | 1.11 | 1.03-1.19 | 0.01 | 1.11 | 1.04-1.19 | <0.01 | 1.16 | 1.06-1.27 | <0.01 | 1.16 | 1.06-1.27 | <0.01 |
| cT |  |  |  |  |  |  |  |  |  |  |  |  |
| T1 | Ref. | Ref. |  | Ref. | Ref. |  | Ref. | Ref. |  | Ref. | Ref. |  |
| T2 | 1.66 | 0.92-3.00 | 0.09 | 0.88 | 0.40-1.94 | 0.75 | 1.67 | 0.93-3.00 | 0.09 | 1.69 | 0.94-3.05 | 0.08 |
| T3 | 4.16 | 1.68-10.28 | <0.01 | 2.41 | 0.96-6.06 | 0.06 | 4.11 | 1.61-10.45 | <0.01 | 5.76 | 2.04-16.27 | <0.01 |

**Supplementary Table I.** Univariable logistic regression analyses assessing the association of SII with adverse surgical features in 214 radio-recurrent PCa treated with SRP

Non-organ confined diseases (pT≥3 and/or pN≥1); Adverse pathology (pT≥3 and/or pN≥1 and/or GS≥8 and/or PSM)

Abbreviations: CI, confidence Interval; DRE, digital rectal examination; GS, Gleason Score; OR, Odds Ratio; PSA, prostate-specific antigen; SRP, salvage radical prostatectomy; SII, Systemic Immune-inflammation Index;
