# Supplementary material for: Role of systemic immune-inflammation index in patients treated with salvage radical prostatectomy
Source: World J Urol. 2021 May 17;39(10):3771–9. doi: 10.1007/s00345-021-03715-4 (PMC8521581; doi:10.1007/s00345-021-03715-4)
Supplement: Supplementary file 2 — Supplementary file2 (DOCX 23 KB) [file 345_2021_3715_MOESM2_ESM.docx]

| Multivariable postoperative models | | | | | | | | | | | | | |
| --- | --- | --- | --- | --- | --- | --- | --- | --- | --- | --- | --- | --- | --- |
|  | BFS | | | MFS | | | CSS | | | OS | | | |
| Characteristic | HR | 95% CI | p-value | HR | 95% CI | p-value | HR | 95% CI | p-value | HR | 95% CI | p-value |  |
| SII (high vs. low) | 0.96 | 0.59-1.57 | 0.870 | 1.17 | 0.39-3.48 | 0.778 | 22.11 | 1.23- 398.12 | **0.036** | 5.98 | 1.67-21.44 | **0.006** |  |
| Age | 1.02 | 0.99-1.06 | 0.234 | 1.04 | 0.97-1.11 | 0.264 | 0.94 | 0.78-1.13 | 0.500 | 1.04 | 0.96-1.14 | 0.324 |  |
| PSA before SRP | 1.02 | 0.99-1.04 | 0.225 | 1.03 | 0.99-1.07 | 0.131 | 1.09 | 0.98-1.21 | 0.135 | 0.99 | 0.94-1.05 | 0.783 |  |
| SRP GS | 1.30 | 1.03-1.64 | **0.029** | 2.10 | 1.33-3.32 | **0.001** | 12.60 | 2.27-69.85 | **0.004** | 3.41 | 1.91-6.09 | **<0.001** |  |
| PSM | 1.42 | 0.84-2.40 | 0.189 | 1.54 | 0.54-4.43 | 0.419 | 0.11 | 0.00-6.97 | 0.299 | 1.60 | 0.49-5.22 | 0.437 |  |
| pT3a | 1.94 | 1.13-3.30 | **0.016** | 2.01 | 0.59-6.79 | 0.263 | 0.97 | 0.06-15.39 | 0.985 | 1.91 | 0.51-7.24 | 0.338 |  |
| pT3b | 1.14 | 0.68-1.91 | 0.621 | 1.78 | 0.62-5.11 | 0.287 | 4.95 | 0.23-104.47 | 0.304 | 2.93 | 0.89-9.66 | 0.077 |  |
| N | 1.74 | 0.97-3.11 | 0.063 | 3.77 | 1.38-11.2 | **0.011** | 0.43 | 0.01-13.09 | 0.632 | 9.29 | 0.97-93.00 | 0.053 |  |
|  | C-index (full model): 72.1  C-index (without SII): 72.1 | | | C-index (full model): 87.9  C-index (without SII): 87.5 | | | C-index (full model): 98.3  C-index (without SII): 98.6 | | | C-index (full model): 92.3  C-index (without SII): 88.6 | | | |

Abbreviations: BFS, biochemical recurrence-free survival, CI, confidence interval; CSS, cancer-specific survival ; GS, Gleason score; HR, hazard ratio; MFS, metastasis-free survival; OS, overall survival; PSA. prostate-specific antigen; PSM, positive surgical margin; SRP, salvage radical prostatectomy; SII, Systemic Immune-inflammation Index
